# Air Quality Is Predictive of Mistakes in Professional Baseball and American Football

**DOI:** 10.3390/ijerph20010542

**Published:** 2022-12-29

**Authors:** Elizabeth C. Heintz, Derek P. Scott, Kolby R. Simms, Jeremy J. Foreman

**Affiliations:** 1 School of Kinesiology, Louisiana State University, Baton Rouge, LA 70803, USA; 2 School of Kinesiology, University of Louisiana at Lafayette, Lafayette, LA 70504, USA

**Keywords:** air pollution, air quality index, climate change, MLB, NFL, baseball, football

## Abstract

Air quality is a growing environmental concern that has implications for human physical and mental health. While air pollution has been linked to cognitive disease progression and declines in overall health, the impacts of air quality on athletic performance have not been extensively investigated. Much of the previous research focused on endurance sports indicates that air quality negatively impacts athletic performance; however, the effects of air quality on non-endurance elite team performance remains largely unknown. The purpose of this study was to examine the impact of air quality on errors committed by Major League Baseball (MLB) teams, interceptions thrown by quarterbacks in the National Football League (NFL), and overall quarterback performance in the NFL. Linear regression analysis was used to determine the impact of the median air quality index (AQI) of counties with MLB and NFL teams on errors, interceptions, and overall quarterback performance of players on those MLB and NFL teams. AQI was a significant positive predictor of errors and interceptions, indicating increased errors and interceptions with decreased air quality. Similarly, quarterback performance was significantly reduced for quarterbacks from teams in counties with worse air quality. These findings suggest that air quality has a significant impact on performance in the MLB and NFL, indicating impairments in physical and cognitive performance in professional athletes when competing in areas with poorer air quality. Hence, it is likely that air quality impacts athletic performance in numerous sports that have not yet been investigated.

## 1. Introduction

Air quality is a major topic relating to increased pollution that has garnered extensive attention due to its impacts on both the environment and human health. In addition to being a major contributor to climate change and overall worsening environmental quality, air quality also affects human physical and mental performance [1,2]. Particulate matter (PM), which are solid particles or liquid droplets in the air, are the primary drivers of environmental and health concerns from worsening air quality [3]. PM_2.5_, small particles ≤2.5 μm in diameter, are inhalable and present the greatest concern for human health with air pollution and can cause cardiopulmonary disorders, airway damage, impairments in immune responses, and increased risk for the development of chronic diseases such as type 2 diabetes [3,4,5]. Hence, air quality has major implications for human health that warrant further investigation to better elucidate the physical and medical consequences of poor air quality.

Impairments in mental and physical performance are the primary concerns of air pollution on human health, and previous research has established both correlative and causal links between PM and performance [6,7,8,9]. For example, PM levels are the primary link between air pollution and cognitive performance [8,10,11]. Poorer air quality has been linked with alterations in decision-making and risk attitude, as well as higher rates of clinical depression [2,12]. Chronic PM exposure appears to have the most severe detrimental effects in elderly adults, as indicated by declines in cognitive performance and accelerated dementia onset [9,13,14]; however, even short-term exposure to higher levels of PM has been shown to decrease executive function in young healthy adults [15]. Air pollution also has both short- and long-term consequences on physical health, with air pollution linked to the increased probability of overall poorer health [7]. Short-term exposure to air pollution decreases hemoglobin levels, with the most severe impacts on men and the elderly [6,16]. High PM levels also increase blood pressure [17], and studies in children have shown that air pollution impairs lung function [18].

While regular exercise conveys immense benefits to health, exercise and physical activity in areas with poorer air quality can actually have detrimental effects on physical health. Acute exercise increases respiration, which in turn increases inhalation and uptake of ultrafine PM at rates proportional to exercise intensity [19]. As a result, PM deposition is nearly quadrupled with exercise [20,21]. Furthermore, PM inhalation is associated with decreased maximal exercise workload and decreased competitive performance, with more detrimental effects seen with increased PM exposure over time [22,23]. Long-term studies have also established that exposure to air pollution decreases lung capacity over time [7], which has serious implications for individuals who perform physical activity in areas with poorer air quality. Recent work has also shown that PM exposure may be linked to alterations in metabolism, specifically in individuals with obesity, further dramatizing impairments in metabolic regulation and increasing risks for other comorbidities [24].

Professional sports offer unique settings to evaluate the effects of air pollution on high-level performance, as elite athletes require the convergence of physical and cognitive performance to excel competitively. Despite this, the impacts of air pollution on physical activity and sports performance have received limited attention relative to the economic and environmental burdens of air pollution, even though large-scale sporting events have been shown to affect both of these factors. For instance, air quality is significantly worse around sporting events to the level where it is often a health concern due to tailgating, cooking, and high traffic [1,25]. Interestingly, while indoor air quality is generally better than outdoor air quality, this is not necessarily the case for sports arenas, particularly in places such as ice rinks where the indoor environment is substantially altered and has been shown to adversely impact athlete health and performance [26,27,28]. Nevertheless, the impacts of outdoor air quality, specifically in areas with greater air pollution, remain a largely understudied area of concern for athlete performance and overall health. Although declining air quality has not significantly discouraged fan attendance at elite athletic events [29,30], there appears to be adverse impacts on athletes when competing in areas with poorer outdoor air quality, as indicated by reductions in high-intensity performance [31]. These studies, in combination with others that have directly shown causative declines in physical health (e.g., [6,7,16,17]), collectively indicate that air quality has impacts on athletic performance, specifically on athletes competing at elite levels where minute differences in physical capacity can determine the outcome of a competition.

To further explore the impacts of air quality on high-level performance and ultimately better elucidate the physical and cognitive impacts of air pollution, we examined the effects of air quality on the performance of professional athletes in two major North American sport leagues: Major League Baseball (MLB) and the National Football League (NFL). The MLB and NFL provide interesting settings to investigate the impacts of outdoor air pollution on elite athletic performance, given that reliable statistics are kept that measure various types of performance. Specifically, errors in MLB and interceptions thrown by quarterbacks in the NFL serve as a marker of performance by indicating the number of major mistakes made, which are suggestive of physical or mental misjudgments. Both mental and physical misjudgments have been linked to poorer air quality. Similarly, overall quarterback performance, as measured by total quarterback rating (QBR) in the NFL, takes into account several measures of quarterback success (e.g., interceptions, passes completed, deep passes), which requires quarterbacks to rely on both their cognitive and physical abilities. Therefore, the purpose of this study is to examine the effects of air quality on errors committed by MLB teams, interceptions thrown by NFL quarterbacks, and overall quarterback performance in the NFL. We hypothesize that an increased number of errors and interceptions will occur among players of teams located in counties with poorer air quality. We also hypothesize that overall quarterback performance will be lower for quarterbacks of teams located in counties with poorer air quality.

## 2. Materials and Methods

Given that we are examining the effects of air quality on team performance in MLB and quarterback performance in the NFL, we use two datasets: a sample of MLB teams and a sample of NFL quarterbacks. Though similar, each of these samples will use a separate regression model. The focus of the MLB team sample is to examine the effects of air quality on errors among the entire team. As the MLB sample does not allow for examining cumulative effects of air quality on performance due to players changing teams from season to season, we use the NFL quarterback sample to examine cumulative effects of air quality on interceptions and overall quarterback performance.

### 2.1. MLB Team Sample

Regression and correlation analyses are used to determine the effects of air quality on professional baseball performance. The study population of MLB teams spans the 1999–2020 MLB seasons. The dependent variable is the number of team errors per game averaged throughout a season (ERRORS). Errors committed by each team are used as a measure of performance, where more errors are indicative of worse performance. Data on MLB errors were collected from baseball-reference.com. The final sample included 632 team-season observations across a 22-year period.

### 2.2. MLB Team Regression Model

The independent variable of interest is median air quality for the year (AIR QUALITY), as determined by the U.S. Environmental Protection Agency (EPA). Air quality data were collected from the EPA (epa.gov) to determine the air quality rating of each home team’s county. The EPA’s Air Quality Index (AQI) defines air quality as follows: Good/Satisfactory: 0–50, Moderate/Acceptable: 51–100, Unhealthy for Sensitive Groups: 101–150, Unhealthy: 151–200, Very Unhealthy: 201–300, Hazardous: >300 [32]; hence, higher AQI is indicative of poorer air quality.

The following control variables are included in the analysis, as they have been shown to impact MLB team performance independent of other factors: MLB season year (YEAR) [33,34], manager tenure with team (MGR TENURE) [35], manager age (MGR AGE) [36], and the natural log of team payroll in millions (LN PAYROLL) [37].

### 2.3. NFL Quarterback Sample

Regression and correlation analyses are also used to examine the effects of air quality on professional football quarterback performance. The study population includes NFL quarterbacks whom began their NFL careers no earlier than 1999 and played for no more than one team per season. The dependent variables are (a) the percent of interceptions thrown per passing attempt (INTERCEPTION%) and (b) total quarterback rating (QBR), as calculated by ESPN Inc (Bristol, CT, USA). While interceptions (i.e., erroneously passing the ball to the opponent) are indicative of poor performance where more interceptions are less desirable, higher QBRs reflect more desirable performance. As QBRs have only been calculated by ESPN Inc. back to the 2006 NFL season, data on NFL interceptions and QBRs are collected for the 2006–2021 NFL seasons from pro-football-reference.com. After using the 2006 season’s dependent variables as predictors of the 2007 season’s dependent variables, the final sample includes 705 quarterback-season observations across a 15-year period.

### 2.4. NFL Quarterback Regression Model

The independent variable of interest is cumulative air quality for a quarterback in a given county (CUM. AIR QUALITY). Cumulative air quality is an average of all the median air quality (i.e., AIR QUALITY) values for a quarterback at every NFL team’s county they played for. While average values are not purely additive, these values are calculated for every NFL quarterback in the sample since they began their NFL careers (no earlier than 1999). As quarterback performance is relative, these relative values reflect the effect of cumulative exposure to poor air quality on interception percentages and total quarterback ratings.

Relatedly, because some quarterbacks have been in the league longer than others, a control variable for NFL experience (NFL EXP) is included in the sample. This variable is similar to MGR TENURE in the MLB errors analysis described earlier in this section. Furthermore, for NFL quarterbacks who often follow the same trajectory to reach the NFL (i.e., high school, then college, then the NFL, without breaks), using NFL EXP as a control variable is similar to using quarterback age as a control variable, which has been carried out in previous examinations of total quarterback performance [38]. Additionally, consistent with the MLB error control variables, as well as previous research examining QBRs, YEAR is included as an independent variable [38]. Prior research examining QBRs also used the previous season’s QBRs as determinants of QBRs in the observed season [38]. Consistent with this previous research, quarterbacks’ interception percentages and QBRs from the season immediately preceding the observed season are included as control variables in the model (INTERCEPTION%_t−1_ and QBR_t−1_). Lastly, to identify starting quarterbacks in the sample from quarterbacks who do not start games (and therefore do not get as much practice time with the other starters on the team), the number of games started by the quarterback in the observed season (GAMES STARTED) is included as a control variable.

### 2.5. Statistical Analyses

Based on the dependent and independent variables for the three models, ordinary least squares (OLS) regression analysis was used to estimate the effect of air quality on the three team performance measures using the following three equations:

*ERRORS = β*_0_*+ β*_1_*(AIR QUALITY) + β*_2_*(YEAR) + β*_3_*(MGR TENURE) + β*_4_*(MGR AGE) + β*_5_*(LN PAYROLL)* (1)

*INTERCEPTION% = β*_0_*+ β*_1_*(AIR QUALITY) + β*_2_*(YEAR) + β*_3_*(NFL EXP) + β*_4_*(INTERCEPTION%_t_*_−1_*) + β*_5_*(GAMES STARTED)* (2)

*QBR = β*_0_*+ β*_1_*(AIR QUALITY) + β*_2_*(YEAR) + β*_3_*(NFL EXP) + β*_4_*(QBR_t_*_−1_*) + β*_5_*(GAMES STARTED)* (3)

## 3. Results

Descriptive statistics for the MLB sample are displayed in Table 1. Per-game errors average over the course of a season ranged from 0.330 to 0.910, with an average of 0.622 errors per game. The average manager tenure was approximately 3 years, and manager age ranged from 35–75 years. Air quality at home stadiums ranged from 38–140, with an average median air quality rating of 61.538.

Table 2 summarizes the correlation matrix of the variables analyzed in the regression. Errors per game are negatively correlated with year, payroll, and manager age, but has a positive correlation with air quality. Conversely, manager tenure with team has no correlation with errors per game.

Regression analysis is presented in Table 3. Team payroll and year count are significant predictors of errors per game, with teams having a lower overall payroll committing more errors per game, while teams generally commit less errors per game each year. Manager age and tenure with team have no significant impact on errors per game. Air quality is also a significant positive predictor of errors per game, with a one-point increase in AQI increasing errors by 0.000993 per game. Therefore, as air quality worsens, so does the number of errors. For example, the AQI in an average county in the sample would be about 67 points better in median AQI than Phoenix, Arizona, which is about 4 standard deviations greater than the mean. Based on this analysis, teams playing in Phoenix would be expected to commit more than 10 additional errors over the course of a 162-game season, potentially reducing overall team standings substantially. Therefore, air quality appears to have a substantial impact on team performance in MLB.

Descriptive statistics for the NFL sample are displayed in Table 4. Interception percentages for quarterbacks range from 0% of passing attempts to one-third of passing attempts, with the average being 2.917% of passing attempts. QBRs range from the lowest possible score of 0 to the highest possible score of 100 but have an average of 48.835. The average cumulative air quality for a quarterback in a given season is 48.871, but ranges from 31.4 to 99.7.

Table 5 summarizes the correlation matrix of the variables analyzed in the regressions for the NFL sample. Interception percentage and QBR are inversely related to each other, as are cumulative air quality and QBR. Interception percentage and cumulative air quality are positively correlated with each other. Consistent with Table 2, over time, in median air quality appears to decrease, as does interception percentage, whereas QBR does not appear to be significantly affected by time in this sample.

Regression analyses for the NFL sample are presented in Table 6. Air quality has a significant negative relationship with QBR (*p* = 0.035) and a marginally significant positive relationship with interception percentage (*p* = 0.059). The coefficient of −0.230 for cumulative air quality on QBR reveals that for every 1-point increase (i.e., worsening) of air quality, there appears to be a 0.230 decrease in QBR. Using the Phoenix example again, playing quarterback for a team in a county like Maricopa County (where Phoenix is located—the home of the Arizona Cardinals) where the median AQI can be about 67 points worse than the average may result in an expected decrease in QBR of about 15.4 points, which is equivalent to about 0.7 standard deviations of QBR. Similarly, interception percentages in Maricopa County could be expected to be about 1.27% higher than the average, which is about 0.4 standard deviations of interception percentages. Therefore, similar to the estimated effect of median air quality on errors in MLB, air quality appears to have a substantial impact on individual quarterback performance in the NFL. Additionally, of interest, time and games started are significantly related to both interception percentages and QBR, whereas NFL playing experience only appears to have a significant effect on interception percentages.

## 4. Discussion

The purpose of this study was to examine the impacts of air quality on errors committed in MLB and NFL quarterback performance. Lower air quality is linked to deficits in physical and cognitive performance, and likely has substantial impacts on elite athlete performance in a variety of sports, though these impacts are largely unexplored. Here, we show that declines in air quality (a) increase the number of errors in a season that are committed by MLB teams, (b) increase the percentage of interceptions thrown by NFL quarterbacks, and (c) decrease overall quarterback performance in the NFL, all of which indicate that poorer air quality negatively impacts performance in elite professional sports that rely heavily on both cognitive and physical abilities.

Regression analysis indicates that errors committed by MLB teams increase by 0.000993 for every point increase in AQI, amounting to a substantial increase in errors over the course of a season for teams playing in areas with worse air quality, thus significantly impacting overall team performance and final rankings. Relatedly, interception percentages increase by 0.019% per point increase in AQI and QBRs decrease by about 0.230 points for every point increase in AQI. These results align with similar effects of air quality on elite athlete performance that were reported in professional soccer. In professional soccer, environmental factors including AQI were linked to worse performance and overall high-intensity work of athletes during a game was decreased in cities with poorer air quality [31,39]. Likewise, professional soccer teams had significantly worse performance when traveling to cities with worse air quality than their home stadium [40], further indicating that alterations in air quality induce noticeable declines in athletic performance. Air quality has also received attention among elite runners [41], where it has been shown that Olympic marathon performance declines with air quality, as indicated by slower finish times in cities with poorer air quality [23]. When individual runners were tracked across multiple races, air quality significantly increased times, showing that individual athletic performance is noticeably altered by changes in air quality [42]. More recently, air quality has been shown to impact elite performance in running distances as short as 5 k [43], further highlighting the effects of air pollution on high-intensity performance. Interestingly, Hodgson et al. highlighted the fact that women’s running times were impacted by higher PM levels in this dataset, whereas men were not [43]. Here, we show that errors increase with higher AQI scores in MLB and the NFL, which currently consist solely of men, thus indicating that the athletic performance of both men and women are potentially impacted by poor air quality, albeit possibly to different extents depending on modality and intensity. Collectively, the results from the present study and previous research suggest that air quality is a significant factor in elite performance. Moreover, these results show that even healthy young men whom regularly exercise are can be adversely affected by air quality that is deemed “acceptable” or “unhealthy for sensitive groups” by the EPA. While these impacts remain poorly understood, it appears that the detrimental effects of air pollution extend beyond the environment and into human physical and mental performance.

Interestingly, the regression results also indicated that errors are decreasing each year in the MLB. While this may be an indication of more elite levels of competition where fewer errors are committed in general [44] or improvements in the number of individual errors committed over time, it is intriguing that the variables ERRORS and YEAR had a significant negative correlation as well.

Team payroll was also a significant predictor of errors, while manager age and tenure with team were not. Increased team pay has been shown to be related to increased performance in the MLB due to being able to afford more talented players, but also due to certain pay structures (e.g., when there is less pay disparity between individual athletes on a team) [45,46], likely due to increased athlete motivation when pay is higher and decreased athlete motivation to perform well when pay is lower.

Contrarily, neither the age of the manager nor the manager’s tenure with the team significantly impacted the number of errors committed. Increased manager experience has been linked to better team outcomes in multiple professional sports, including the MLB, while managerial turnover often causes decreases in team performance [35,47,48]. While this is not supported by the current analysis, it is possible that managerial age or tenure did have impacts on MLB team performance during the sample period, just on different variables other than team errors. For instance, it is possible that managers may focus more on batting and pitching than fielding, resulting in more wins, increased batting averages, or improved earned run averages based on increases in managerial tenure. Contrarily, it is possible that the lack of impact of manager characteristics on errors is reflective of an insignificant effect of managers on overall team performance in the MLB. Similarly, NFL playing experience for quarterbacks does not seem to significantly improve QBRs. However, NFL playing experience does appear to be correlated with reductions in interceptions, which may be a result of learning on the job or due to poorer performers not lasting as long in the NFL and exiting earlier than higher-performing quarterbacks whom throw fewer interceptions per passing attempt.

Although the present analysis shows that there is a significant impact of air quality on errors committed in professional baseball and American football, athletic performance is multifactorial. While the present study accounts for numerous factors that have been shown to impact performance in MLB and the NFL, such as experience, there are additional factors that may also impact athlete performance that were not included in the present model. These include environmental factors such as atmospheric pressure and temperature, as well as individual and team-based factors such as the distance traveled [49] and sleep quality [50]. While these limitations warrant further investigation to fully determine the impacts of air quality on athletic performance with the inclusion of these factors, the current model shows that there is a significant negative impact on errors committed in the MLB with poorer air quality, indicating that air quality does have substantial impacts on performance in professional baseball, and likely other sports as well.

## 5. Conclusions

The results of this study indicate that AQI is a significant predictor of errors in the MLB, interceptions in the NFL, and quarterback performance in the NFL, indicating that more mistakes are committed, and performance declines, in areas with worse air quality. To our knowledge, this is the first evidence that air quality has a negative impact on MLB or NFL performance, specifically on factors that are related to both high cognitively and physically demanding activities such as errors in MLB and interceptions and overall quarterback performance in the NFL. While the majority of previous research related to air quality and performance is focused on endurance sports, here, we provide evidence that air quality has negative impacts on performance in sports dominated by short, fast plays, even for healthy young men. These findings indicate that air quality has effects that extend beyond climate change and into human health and performance, most of which are just beginning to be explored. These results have implications for MLB and NFL athletes and teams, as air quality likely impacts individual performance and overall team outcomes. Furthermore, these findings are relevant to all athletes competing in numerous areas with varying air quality, as this likely impacts their performance in ways that are poorly understood. Moreover, fans within cities with professional sports teams may elect to take efforts to improve air quality in order to see their local sports teams gain a competitive advantage. Similarly, results may also be generalizable to other populations who want to maintain physical and cognitive abilities for themselves or their children, and therefore, may also seek to improve the air they breathe within their counties and households. Based on these findings, future research should investigate the causative factors of poor air quality on declines in athlete performance across multiple modalities to better understand the implications of air quality on all aspects of human athletic performance as well as other cognitively and physically demanding activities.

## Figures and Tables

**Table 1:** Descriptive statistics for MLB sample.

Variable	Mean	Std. Dev.	Min.	Max.
ERRORS	0.622	0.105	0.330	0.910
YEAR	10.576	6.329	0	21
MGR TENURE	3.044	3.439	0	19
MGR AGE	53.470	7.507	35	75
LN PAYROLL	4.436	0.502	2.710	5.54
AIR QUALITY	61.538	14.626	38	140

**Table 2:** Correlation matrix of regression variables for MLB sample.

	ERRORS	YEAR	MGR TENURE	MGR AGE	LN PAYROLL
YEAR	−0.356 ***				
MGR TENURE	−0.054	0.004			
LN PAYROLL	−0.376 ***	0.577 ***	0.144 ***		
MGR AGE	−0.122 ***	0.067 *	0.312 ***	0.209 ***	
AIR QUALITY	0.243 ***	−0.490 ***	0.006	−0.177 ***	−0.127 ***

Note. One-tailed *p*-values presented for hypothesized AIR QUALITY independent variable with ERRORS dependent variable, otherwise, two-tailed *p*-values presented. * *p* < 0.1, *** *p* < 0.01.

**Table 3:** Regression results of air quality as a predictor of MLB errors.

Variable	Coefficient	Std. Err.	*p*-Value
YEAR	−0.002	0.001	0.041
MGR TENURE	0.001	0.001	0.618
LN PAYROLL	−0.063	0.011	<0.001
MGR AGE	−0.001	0.001	0.175
AIR QUALITY	0.001	<0.001	0.005
Constant	0.900	0.055	<0.001

Note. One-tailed *p*-values presented for hypothesized AIR QUALITY independent variable, otherwise, two-tailed *p*-values presented.

**Table 4:** Descriptive statistics for NFL sample.

Variable	Mean	Std. Dev.	Min.	Max.
INTERCEPTION%	2.917	3.239	0	33.3
QBR	48.835	21.584	0	100
YEAR	15.167	4.326	8	22
NFL EXP	5.627	3.929	1	21
GAMES STARTED	8.526	6.515	0	17
CUM. AIR QUALITY	48.871	8.083	31.4	99.7

**Table 5:** Correlation matrix of regression variables for MLB sample.

	INTERCEPTION%	QBR	YEAR	NFL EXP	GAMES STARTED
QBR	−0.308 ***				
YEAR	−0.132 ***	−0.010			
NFL EXP	−0.108 ***	0.147 ***	0.229 ***		
GAMES STARTED	−0.186 ***	0.441 ***	0.059	0.140 ***	
CUM. AIR QUALITY	0.060 *	−0.058 *	−0.196 ***	0.011	0.038

Note. One-tailed *p*-values presented for hypothesized AIR QUALITY independent variable with dependent variables INTERCEPTION% and QBR, otherwise, two-tailed *p*-values presented. * *p* < 0.1, *** *p* < 0.01.

**Table 6:** Regression results of air quality as a predictor of interceptions and QBR.

	INTERCEPTION%		QBR	
Variable	Coefficient	*p*-Value	Coefficient	*p*-Value
INTERCEPTION%_t−1_	0.019	0.613	-	-
QBR_t−1_	-	-	−0.004	0.932
YEAR	−0.072	0.007	−0.430	0.050
NFL EXP	−0.049	0.037	0.287	0.219
GAMES STARTED	−0.085	<0.001	1.229	<0.001
AIR QUALITY	0.018	0.059	−0.230	0.035
Constant	0.900	<0.001	53.506	<0.001

Note. One-tailed *p*-values presented for hypothesized AIR QUALITY independent variable, otherwise two-tailed *p*-values presented.

## Data Availability

Data regarding baseball performance, managers, and payrolls were obtained from baseball-reference.com. Data on American football quarterback performance were collected from pro-football-reference.com. Data on air quality were collected from epa.gov.
